# Predictors of urinary function recovery after laparoscopic and robot-assisted radical prostatectomy

**DOI:** 10.1590/S1677-5538.IBJU.2022.0362

**Published:** 2022-11-15

**Authors:** Kyohei Hakozaki, Toshikazu Takeda, Yota Yasumizu, Nobuyuki Tanaka, Kazuhiro Matsumoto, Shinya Morita, Takeo Kosaka, Ryuichi Mizuno, Hiroshi Asanuma, Mototsugu Oya

**Affiliations:** 1 Keio University School of Medicine Department of Urology Tokyo Japan Department of Urology, Keio University School of Medicine, Shinanomachi, Shinjuku-ku, Tokyo, Japan

**Keywords:** Prostatic Neoplasms, Robotic Surgical Procedures, Urinary Incontinence

## Abstract

**Introduction:**

Even in the era of laparoscopic radical prostatectomy (LRP) and robot-assisted laparoscopic radical prostatectomy (RALP), we sometimes encounter patients with severe urinary incontinence after surgery. The aim of the present study was to identify predictors of urinary continence recovery among patients with urinary incontinence immediately after surgery (UIIAS).

**Materials and Methods:**

We identified 274 patients with clinically localized prostate cancer who underwent LRP and RALP between 2011 and 2018. UIIAS was defined as a urine loss ratio > 0.15 on the first day of urethral catheter removal. Urinary continence recovery was defined as using ≤ 1 pad/day one year after surgery. In the present study, we evaluated factors affecting urinary function recovery one year after surgery among patients with urinary incontinence immediately after LRP and RALP.

**Results:**

UIIAS was observed in 191 out of 274 patients (69.7%). A multivariate analysis identified age (< 65 years, p = 0.015) as an independent predictor affecting immediate urinary continence. Among 191 incontinent patients, urinary continence one year after surgery improved in 153 (80.1%). A multivariate analysis identified age (< 65 years, p = 0.003) and estimated blood loss (≥ 100 mL, p = 0.044) as independent predictors affecting urinary continence recovery one year after surgery.

**Conclusion:**

The present results suggest that younger patients and patients with higher intraoperative blood loss recover urinary continence one year after surgery even if they are incontinent immediately after surgery.

## INTRODUCTION

Prostate cancer (PCa) is one of the most prevalent cancers in men. Radical prostatectomy (RP) is a radical treatment for localized PCa; however, it is associated with complications including postoperative urinary incontinence. Laparoscopic RP (LRP) was developed 25 years ago, and robot-assisted laparoscopic RP (RALP) rapidly became the standard surgery for PCa. Even in the era of LRP and RALP, we sometimes encounter patients with severe postoperative urinary incontinence. A recent review revealed no significant differences in urinary continence between RALP and open retropubic RP 12 months after surgery (1). Ficarra et al. reported no functional superiority among surgical methods (2). Another study revealed no significant differences in the proportion of pad-free patients among open retropubic RP, LRP, and RARP groups during a one-year follow-up after RP (3). Therefore, postoperative urinary incontinence is still a serious complication for patients who undergo LRP and RALP. Since postoperative urinary incontinence has a significant impact on quality of life (4, 5), many studies have attempted to reduce urinary incontinence by using modified surgical techniques (6, 7). Despite these attempts, the prevalence of postoperative urinary incontinence has risen, paralleling the increase in the number of surgeries performed every year (8), and thus, postoperative urinary incontinence remains an important issue.

In a recent meta-analysis, continence rates twelve months after LRP ranged between 66 and 95% (2); however, those of early urinary incontinence are markedly higher. One study reported that the likelihood of a patient requiring pads after surgery was typically 70 - 80% at 6 weeks and 50 - 60% at three months (9). Although the majority of these patients regained urinary continence (using ≤ 1 pad/day) by one year (10), the patients are anxious about when their continence will improve.

Multiple factors are involved in the recovery of urinary continence after RP, including the chronological, anatomical, and oncological conditions of patients, and the surgical techniques and modifications employed (6, 11, 12). However, to the best of our knowledge, the predictors of urinary continence recovery among patients with urinary incontinence immediately after RP have not yet been elucidated in detail. Factors that improve urinary incontinence one year after surgery among patients with urinary incontinence immediately after surgery (UIIAS) currently remain unknown. We hypothesized that not only the preoperative patient background, but also intraoperative factors, such as surgical techniques, may influence urinary continence one year after surgery. Therefore, the aim of the present study was to identify predictors of urinary continence recovery among patients with urinary incontinence immediately after LRP and RALP.

## MATERIALS AND METHODS

### Enrollment

After approval by the Institutional Review Board (IRB number; 20160084), we identified 442 patients with clinically localized PCa at Keio University Hospital (Tokyo, Japan) between December 2011 and May 2018. We excluded patients with urinary leakage at cystography (CG) after surgery (N = 6) and urinary retention after removal of the urethral catheter (N = 13), because we were unable to calculate their urine volume. We also excluded patients who underwent salvage radiation therapy within one year of surgery (N = 8), and those with missing data, such as the lack of a urine loss ratio (ULR) description (N = 141). Therefore, 274 patients who underwent LRP (N = 143) and RALP (N = 131) were included.

We used ULR to evaluate UIIAS. ULR is defined as the weight of urine loss in a pad divided by the daily micturition volume (13, 14). Ates et al. reported that the first day on which ULR exceeded 15% correlated with an increased risk of urinary incontinence (13). Therefore, UIIAS was defined as ULR > 0.15 on the first day of urethral catheter removal in the present study. Urinary continence recovery was defined as using ≤ 1 pad/day one year after surgery; this definition has been used in previous studies (15, 16). Nurses in the urology ward provide instructions on pelvic floor muscle training (PFMT) before surgery and initiate PFMT after the removal of the urethral catheter in our hospital.

## SURGICAL TECHNIQUE

LRP and RALP were performed under general anesthesia and patient-controlled intravenous analgesia. We performed LRP in a supine position and RALP was mainly performed using the same degree of Trendelenburg tilt (25°). In the case of glaucoma and stroke, we performed RALP in a supine position to prevent increases in intraocular pressure and intracranial pressure during surgery due to the prolonged use of a steep Trendelenburg position. LRP was performed using an extraperitoneal five-port approach, and the carbon dioxide insufflation pressure was typically maintained at 10 mmHg during surgery. RALP was conducted by the same surgical team using the da Vinci^®^ Xi surgical system. RALP was performed using both transperitoneal and extraperitoneal approaches; however, the transperitoneal approach was more frequent as it provides a wider space to work. In the present study, 117 out of 131 patients underwent RALP with a transperitoneal approach. The remaining 14 patients underwent RALP with an extraperitoneal approach due to a history of abdominal surgery, glaucoma, and stroke. RALP was performed using six ports, and the carbon dioxide insufflation pressure was typically maintained at 10 mmHg during surgery. Pelvic lymph node dissection including the bilateral internal iliac, external iliac and obturator lymph nodes was performed in both LRP and RALP. The dorsal venous complex (DVC) was processed by cold incision and selective sutures in both LRP and RALP. In either method, hemostasis by thermal coagulation was minimized. We also performed preservation of the urethral length and posterior reconstruction of the rhabdosphincter (Rocco's stitch) before vesicourethral anastomosis in both LRP and RALP. On the other hand, we did not perform bladder neck preservation, puboprostatic ligaments sparing the preservation of the endopelvic fascia, Retzius sparing, or complete anterior preservation. Nerve sparing was conducted according to the location of the lesion and requests by patients (Supplementary Table-1). After vesicourethral anastomosis, the integrity of the anastomosis was tested by instilling 100 mL of saline into the bladder with a urethral catheter. At the end of surgery, a 5-mm drainage tube was placed in the vesicourethral anastomotic part. We estimated blood loss based on the observation of the weight of surgical gauze used and the amount of fluid in the suction device during surgery. CG was performed on the fourth postoperative day (POD), on average, and the urethral catheter was removed if there was no urinary leakage.

**Supplementary Table 1:** Preservation procedure in the present study.

Performed	Not performed	Depending on the case
urethral length preservation	bladder neck preservation	nerve sparing
selective suturing of dorsal venous complex	puboprostatic ligaments sparing preservation of the endopelvic fascia			
	complete anterior preservation
	Retzius sparing

### Statistical analysis

The relationships between clinicopathological backgrounds and urinary continence one year after surgery or first day ULR were analyzed by the chi-squared test or Mann-Whitney U test. Independent variables examined in the present study were patient age (< 65 years vs. ≥ 65 years), body mass index (BMI) (≤ 22 kg/m2 vs. > 22 kg/m2), the presence of diabetes (DM) (yes vs. no), prostate specific antigen (PSA) level (≤ 10 ng/mL vs. > 10 ng/mL), prostate volume (≤ 30 mL vs. > 30 mL), clinical T stage (≤ T2 vs. ≥ T3), Gleason score (< 7 vs. ≥ 7), surgical method (LRP vs. RALP), nerve-sparing (including unilateral or bilateral) or not (yes vs. no), estimated blood loss (EBL) (< 100 mL vs. ≥ 100 mL), the surgical time (< 200 minutes vs. ≥ 200 minutes), and the duration of urethral catheterization (until 4 POD vs. over 5 POD). Univariate and multivariate analyses that predict urinary continence one year after surgery or first day ULR were performed using logistic regression models.

All reported p values were two-sided, and significance was set at p < 0.05. These analyses were performed with SPSS ver. 25.0 statistical software package (IBM Corp., Armonk, NY, USA).

The Ethics Committee of the Keio University School of Medicine waived the requirement for informed consent for this study.

## RESULTS

Among 274 patients, median age at the time of surgery was 67 (47 - 76) years, median BMI was 23.7 (15.2 - 37.7) kg/m2, the median PSA value was 6.9 (3.8 - 72.9) ng/mL, the median prostate volume was 30.0 (10.3 - 80.4) mL, the median volume of EBL was 150 (30 - 2250) mL, the median surgical time was 199 (70 - 459) minutes, and median first day ULR was 0.31 (0.00 - 1.00) (all values are medians (range) (Table-1).

**Table 1:** Clinicopathological backgrounds in overall patients.

Characteristic		Total	first day ULR ≤ 0.15	first day ULR > 0.15	p value	Continence	Incontinence	p value
No. of patients		274	83	191		233	41	
**Age (years)**					**0.027**			**< 0.001**
	Median	67	65	67		66	69	
	Range	47 - 76	47 - 76	48 - 76		47 - 76	60 - 75	
**BMI (kg/m^2^)**					**0.755**			**0.494**
	Median	23.7	23.4	23.8		23.6	24.0	
	Range	15.2 - 37.7	15.2 - 37.7	15.8 - 33.9		15.2 - 37.7	18.3 - 30.8	
**DM**					**0.421**			**0.498**
	Yes	24	9	15		20	4	
	No	250	74	176		213	37	
**PSA (ng/mL)**					**0.354**			**0.387**
	Median	6.9	6.3	7.0		6.8	7.5	
	Range	3.8 - 72.9	4.0 - 47.9	3.8 - 72.9		4.0 - 72.9	3.8 - 27.0	
**Prostate volume (mL)**					**0.265**			**0.792**
	Median	30.0	29.5	30.2		29.8	30.4	
	Range	10.3 - 80.4	15.1 - 61.4	10.3 - 80.4		10.3 - 80.4	15.3 - 67.1	
**Clinical T stage (n)**					**0.752**			**0.387**
	≤ T2	253	76	177		216	37	
	≥ T3	21	7	14		17	4	
**Gleason score (n)**					**0.420**			**0.572**
	< 7	33	8	25		28	5	
	≥ 7	241	75	166		205	36	
**Surgical method (n)**					**0.218**			**0.136**
	LRP	143	48	95		126	17	
	RALP	131	35	96		107	24	
**Nerve sparing (n)**					**0.640**			**0.723**
	Performed	61	17	44		51	10	
	Not performed	213	66	147		182	31	
	Blood loss (mL)				**0.577**			**0.208**
	Median	150	125	150		151	142	
	Range	0 - 2250	0 - 800	0 - 2250		0 - 2250	0 - 600	
**Surgical time (minutes)**					**0.472**			**0.122**
	Median	199	191	200		195	210	
	Range	70 - 459	91 - 314	70 - 459		70 - 459	115 - 420	
**Urethral catheter removal (POD)**					**0.079**			**0.166**
	Median	4	4	4		4	4	
	Range	2 - 9	2 - 6	2 - 9		2 - 9	2 - 6	
**First day ULR**								**< 0.001**
	Median	0.31	-	-		0.28	0.52	
	Range	0.00 - 1.00	-	-		0.00 - 1.00	0.07 - 1.00	

BMI = body mass index; DM = diabetes mellitus; LRP = laparoscopic radical prostatectomy; POD = postoperative day; PSA = prostate specific antigen; RALP = robot-assisted laparoscopic radical prostatectomy; ULR = urine loss ratio

Urinary continence one year after surgery was observed in 233 out of 274 patients (85.0%). A multivariate analysis identified age (< 65 years, p = 0.002) and first day ULR (≤ 0.15, p = 0.005) as independent predictors affecting urinary continence one year after surgery in all patients. Other clinical and pathological features were not associated with urinary continence one year after surgery (Table-2).

**Table 2:** Predicting of urinary continence at one year after surgery.

Characteristic		Total	Univariate					Multivariate		
			Continence		Incontinence		p value	OR	95% CI	p value
No. of patients		274	233	85.0	41	15.0				
**Age**							**0.001**	**4.113**	**1.646 - 10.275**	**0.002**
	< 65 years	90	87	37.3	3	7.3				
	≥ 65 years	184	146	62.7	38	92.7				
**BMI**							**0.854**			
	≤ 22 kg/m^2^	70	60	25.8	10	24.4				
	> 22 kg/m^2^	204	173	74.2	31	75.6				
**DM**							**0.807**			
	Yes	24	20	8.6	4	9.8				
	No	250	213	91.4	37	90.2				
**PSA**							**0.686**			
	≤ 10 ng/mL	207	175	75.1	32	78.0				
	> 10 ng/mL	67	58	24.9	9	22.0				
**Prostate volume**							**0.971**			
	≤ 30 mL	153	130	55.8	23	56.1				
	> 30 mL	121	103	44.2	18	43.9				
**Clinical T stage**							**0.586**			
	≤ T2	253	216	92.7	37	90.2				
	≥ T3	21	17	7.3	4	9.8				
**Gleason score**							**0.974**			
	< 7	33	28	12.0	5	12.2				
	≥ 7	241	205	88.0	36	87.8				
**Surgical method**							**0.139**			
	LRP	143	126	54.1	17	41.5				
	RALP	131	107	45.9	24	58.5				
**Nerve sparing**							**0.723**			
	Performed	61	51	21.9	10	24.4				
	Not performed	213	182	78.1	31	75.6				
**Blood loss**							**0.083**			
	< 100 mL	82	65	27.9	17	41.5				
	≥ 100 mL	192	168	72.1	24	58.5				
**Surgical time**							**0.295**			
	< 200 minutes	141	123	52.8	18	43.9				
	≥ 200 minutes	133	110	47.2	23	56.1				
**Urethral catheter removal**							**0.147**			
	Until 4 POD	223	193	82.8	30	73.2				
	Over 5 POD	51	40	17.2	11	26.8				
**First day ULR**							**0.002**	**5.710**	**1.690 - 19.292**	**0.005**
	≤ 0.15	83	80	34.3	3	7.3				
	> 0.15	191	153	65.7	38	92.7				

BMI = body mass index; CI = confidence interval; DM = diabetes mellitus; LRP = laparoscopic radical prostatectomy; OR = odds ratio; POD = postoperative day; PSA = prostate specific antigen; RALP = robot-assisted laparoscopic radical prostatectomy; ULR = urine loss ratio / All values are frequency (proportion).

Among 274 patients, 83 (30.3%) were continent immediately after surgery. A multivariate analysis identified age (< 65 years, p = 0.015) as an independent predictor affecting urinary continence immediately after surgery. Other clinical and pathological features were not associated with urinary continence immediately after surgery (Table-3).

**Table 3:** Predicting of the first day ULR ≤ 0.15.

Characteristic		Univariate					Multivariate		
		first day ULR ≤ 0.15		first day ULR > 0.15		p value	OR	95% CI	p value
No. of patients		83	30.3	191	69.7				
**Age**						**0.015**	**1.943**	**1.137 - 3.322**	**0.015**
	< 65 years	36	43.4	54	28.3				
	≥ 65 years	47	56.6	137	71.7				
**BMI**						**0.951**			
	≤ 22 kg/m^2^	21	25.3	49	25.7				
	> 22 kg/m^2^	62	74.7	142	74.3				
**DM**						**0.423**			
	Yes	9	10.8	15	7.9				
	No	74	89.2	176	92.1				
**PSA**						**0.315**			
	≤ 10 ng/mL	66	79.5	141	73.8				
	> 10 ng/mL	17	20.5	50	26.2				
**Prostate volume**						**0.219**			
	≤ 30 mL	51	61.4	102	53.4				
	> 30 mL	32	38.6	89	46.6				
**Clinical T stage**						**0.752**			
	≤ T2	76	91.6	177	92.7				
	≥ T3	7	8.4	14	7.3				
**Gleason score**						**0.422**			
	< 7	8	9.6	25	13.1				
	≥ 7	75	90.4	166	86.9				
**Surgical method**						**0.219**			
	LRP	48	57.8	95	49.7				
	RALP	35	42.2	96	50.3				
**Nerve sparing**						**0.641**			
	Performed	17	20.5	44	23.0				
	Not performed	66	79.5	147	77.0				
**Blood loss**						**0.535**			
	< 100 mL	27	32.5	55	28.8				
	≥ 100 mL	56	67.5	136	71.2				
**Surgical time**						**0.735**			
	< 200 minutes	44	53.0	97	50.8				
	≥ 200 minutes	39	47.0	94	49.2				
**Urethral catheter removal**						**0.409**			
	Until 4 POD	70	84.3	153	80.1				
	Over 5 POD	13	15.7	38		19.9			

BMI = body mass index; CI = confidence interval; DM = diabetes mellitus; LRP = laparoscopic radical prostatectomy; OR = odds ratio; POD = postoperative day; PSA = prostate specific antigen; RALP = robot-assisted laparoscopic radical prostatectomy; ULR = urine loss ratio / All values are frequency (proportion).

We then evaluated the remaining 191 patients who were incontinent immediately after surgery. Among them, 153 patients (80.1%) showed improved urinary continence one year after surgery, while 38 (19.9%) remained incontinent. A multivariate analysis identified age (< 65 years, p = 0.003) and EBL (≥ 100 mL, p = 0.044) as independent predictors affecting urinary continence recovery one year after surgery. Other factors were not independent predictors of urinary continence recovery among patients with UIIAS (Table-4).

**Table 4:** Predicting of urinary continence at one year after surgery in patients with UIIAS.

Characteristic		Total	Univariate					Multivariate		
			continence		incontinence		p value	OR	95% CI	p value
No. of patients		191	153	80.1	38	19.9				
**Age**							**< 0.001**	**9.479**	**2.181 - 41.196**	**0.003**
	< 65 years	54	52	34.0	2	5.3				
	≥ 65 years	137	101	66.0	36	94.7				
**BMI**							**0.756**			
	≤ 22 kg/m^2^	49	40	26.1	9	23.7				
	> 22 kg/m^2^	142	113	73.9	29	76.3				
**DM**							**0.496**			
	Yes	176	142	92.8	34	89.5				
	No	15	11	7.2	4	10.5				
**PSA**							**0.696**			
	≤ 10 ng/mL	141	112	73.2	29	76.3				
	> 10 ng/mL	50	41	26.8	9	23.7				
**Prostate volume**							**0.797**			
	≤ 30 mL	102	81	52.9	21	55.3				
	> 30 mL	89	72	47.1	17	44.7				
**Clinical T stage**							**0.403**			
	≤ T2	66	54	35.3	12	31.6				
	≥ T3	125	99	64.7	26	68.4				
**Gleason score**							**0.989**			
	< 7	25	20	13.1	5	13.2				
	≥ 7	166	133	86.9	33	86.8				
**Surgical method**							**0.295**			
	LRP	95	79	51.6	16	42.1				
	RALP	96	74	48.4	22	57.9				
**Nerve sparing**							**0.746**			
	Performed	44	36	23.5	8	21.1				
	Not performed	147	117	76.5	30	78.9				
**Blood loss**							**0.046**	**2.207**	**1.020 - 4.777**	**0.044**
	< 100 mL	55	39	25.5	16	42.1				
	≥ 100 mL	136	114	74.5	22	57.9				
**Surgical time**							**0.406**			
	< 200 minutes	97	80	52.3	17	44.7				
	≥ 200 minutes	94	73	47.7	21	55.3				
**Urethral catheter removal**							**0.271**			
	Until 4 POD	153	125	81.7	28	73.7				
	Over 5 POD	38	28	18.3	10	26.3				

BMI = body mass index; CI = confidence interval; DM = diabetes mellitus; LRP = laparoscopic radical prostatectomy; OR = odds ratio; POD = postoperative day; PSA = prostate specific antigen; RALP = robot-assisted laparoscopic radical prostatectomy; UIIAS = urinary incontinence immediately after surgery; ULR = urine loss ratio

All values are frequency (proportion).

## DISCUSSION

Even in the era of LRP and RALP, urinary incontinence after RP remains a distressing complication that affects postoperative quality of life (5, 7, 11, 12). The precise etiology of postoperative urinary incontinence is unclear (17, 18). However, previous studies have suggested selective suture ligation of the DVC to preserve the rhabdosphincter and underlying neurovascular components, which may improve the recovery of urinary continence (17). Other studies revealed that Retzius-sparing RALP contributed to postoperative urinary continence (19, 20). These findings indicate the importance of reducing the complications of postoperative urinary incontinence by selecting the optimal surgical procedure. While most patients with UIIAS will recover their urinary function by one year (2, 3, 9, 10), those who do not may require additional medical treatment or surgery (21). Therefore, we need to identify patients at a higher risk of postoperative urinary incontinence even one year after surgery among those with UIIAS.

The present results revealed two predictors of urinary function recovery. Age was an independent predictor of both immediate continence and the recovery of urinary continence. According to previous studies, increased age is associated with an increased prevalence of postoperative incontinence (18, 22, 23). The mechanism underlying age-related postoperative urinary incontinence currently remains unclear (17, 18). Strasser et al. noted age-dependent decreases in the density of striated muscle cells in necropsies, and concluded that this may be the main reason for the higher incidence of urinary incontinence with increasing age (24). Other studies have suggested that the natural decrease in rhabdosphincter cells with aging contributes to the increasing incidence of stress incontinence with age, and that this process may be further accelerated by the surgical trauma of RP (18). They also speculated that the healing process leading to the restitution of normal function was less successful with increasing age. Many clinical and animal studies at the cellular and molecular levels examined age-related changes and delays in wound healing (25). Age is a risk factor for impaired wound healing. Therefore, young people are unlikely to have UIIAS, and even if they do, the repair of sphincter tissue is likely to occur. This is considered to improve urinary function one year after surgery. The present results on age and urinary continence are supported by previous findings.

High EBL (≥ 100 mL) at LRP and RALP was identified as an independent predictor of urinary continence recovery. This result is considered to be related to the content of the surgical technique. In a previous study that evaluated the relationship between EBL and postoperative urinary incontinence, blood loss did not affect continence rates 24 months after surgery (26). Preisser et al. recently reported on the relationship between EBL during RP and postoperative urinary function (27). They identified 2,720 patients who underwent RALP between 2009 and 2015, and defined EBL of 150 mL or less as low, EBL exceeding 400 mL as high and 150 - 400 mL as medium. High EBL was an independent predictor for seven days of incontinence in patients undergoing RALP. However, high EBL at RALP was not an independent predictor of incontinence three months or one year after surgery (27). They considered one of the biological reasons for these findings to blood loss being a recoverable factor within the normal hematopoietic capacity. Furthermore, high EBL increases the risk and area of coagulation hemostasis, which may be an exacerbating factor in UIIAS. Lei et al. demonstrated that the athermal procedure of DVC had a positive effect on postoperative urinary continence (17). Therefore, minimal coagulation hemostasis during surgery may lead to an increase in EBL, and this has the advantage of improving urinary continence. If the increases in EBL are not due to minimal coagulation hemostasis, but mere carelessness at surgery, urinary incontinence may not recover immediately or one year after surgery. As described in the Materials and Methods section, we use minimal coagulating hemostasis with cold incision and selective suturing to treat DVC. The results obtained revealed that first day ULR did not deteriorate even in high EBL patients. In contrast, urinary incontinence improved one year after surgery in high EBL patients, as reported by Preisser et al. (27). The results of the present study suggest that minimal coagulating hemostasis improved urinary function within one year.

We did not identify any influence of BMI or prostate volume on postoperative urinary continence. Although previous studies reported that a lower BMI and smaller prostate volume were associated with the better recovery of urinary continence (11, 12), these findings are controversial in terms of the relationship between obesity and urinary incontinence after RP (28). Another study showed that the influence of prostate volume on continence varied (29).

The present study has some limitations. This was a single institution study, and the cohort was small. Further studies with larger sample sizes are needed to confirm the predictors of urinary function recovery among patients with UIIAS. Furthermore, this study was not conducted as a single-surgeon series. Despite all surgeons using virtually the same techniques, slight differences in procedures among surgeons may have affected postoperative urinary continence. In addition, since this was a retrospective study, we were unable to analyze possible predictors, such as the preoperative urinary condition. Despite these limitations, the present study is original in that it focused on patients with UIIAS. Moreover, it is important to note that intraoperative techniques, such as minimal coagulating hemostasis and cold incision, may have contributed to improvements in urinary incontinence.

## CONCLUSION

The present study revealed that a young age and higher intraoperative blood loss at LRP and RALP are predictors of urinary function recovery among patients with UIIAS. The results of the present study may help in explaining to patients with UIIAS the importance of surgical techniques, such as minimal coagulation hemostasis.

## ABBREVIATIONS

LRP =: laparoscopic radical prostatectomy
RALP =: robot-assisted laparoscopic radical prostatectomy
UIIAS =: urinary incontinence immediately after surgery
PCa =: prostate cancer
RP =: radical prostatectomy
CG =: cystography
DVC =: dorsal vein complex
POY =: postoperative year
POD =: postoperative day
ULR =: urine loss ratio
BMI =: body mass index
DM =: diabetes mellitus
PSA =: prostate specific antigen
EBL =: estimated blood loss
